# New Developments in T Cell Immunometabolism and Implications for Cancer Immunotherapy

**DOI:** 10.3390/cells11040708

**Published:** 2022-02-17

**Authors:** Nathaniel Oberholtzer, Kristen M. Quinn, Paramita Chakraborty, Shikhar Mehrotra

**Affiliations:** Department of Surgery, Medical University of South Carolina, Charleston, SC 29425, USA; quinnkr@musc.edu (K.M.Q.); chakrabp@musc.edu (P.C.)

**Keywords:** immunotherapy, cancer, T cell metabolism, immunometabolism, antitumor metabolism

## Abstract

Despite rapid advances in the field of immunotherapy, the elimination of established tumors has not been achieved. Many promising new treatments such as adoptive cell therapy (ACT) fall short, primarily due to the loss of T cell effector function or the failure of long-term T cell persistence. With the availability of new tools and advancements in technology, our understanding of metabolic processes has increased enormously in the last decade. Redundancy in metabolic pathways and overlapping targets that could address the plasticity and heterogenous phenotypes of various T cell subsets have illuminated the need for understanding immunometabolism in the context of multiple disease states, including cancer immunology. Herein, we discuss the developing field of T cell immunometabolism and its crucial relevance to improving immunotherapeutic approaches. This in-depth review details the metabolic pathways and preferences of the antitumor immune system and the state of various metabolism-targeting therapeutic approaches.

## 1. Introduction

Since Lloyd J Old’s original proposal that specific antigens were associated with tumors [1], the field of cancer immunotherapy has made significant advances to improve efficacy, including the use of high-dose IL-2 therapy, cloning tumor antigens, and identifying tumor-reactive T cells [2,3,4,5,6,7,8]. Soon after, the cloning of the T cell receptor (TCR) and demonstration of its successful engineering in T cells [9] led to the revolutionizing approach of transferring patient T cells that had been genetically modified to recognize tumor antigens [10,11]. These efforts have been boosted by the use of chimeric antigen receptors (CARs) [12] and blocking co-inhibitory receptors on T cells for maintaining T cell effector function and persistence in vivo [13].

While the above approaches, which engage antitumor T cells to control tumors, have been reasonably successful, especially for hematological malignancies [14], the obstacles posed by the suppressive tumor microenvironment (which either renders the transferred T cells dysfunctional or reduces their viability) have led to non-reproducible effects in terms of tumor control [15]. Thus, strategies to understand how durable tumor control can be achieved by programming tumor-reactive T cells are being tested. One important strategy at the forefront of immunotherapy research is the targeting of energy metabolism pathways so that the antitumor T cells can compete with glycolytic tumors and maintain their effector and cytolytic activity in vivo [16,17,18,19,20,21,22,23,24,25]. The metabolic fitness of antitumor T cells can be improved by enhancing their mitochondrial metabolism and by reducing their dependence on glycolysis [22,26]. In addition, lipolysis, which fuels mitochondrial oxidative phosphorylation (OXPHOS) and/or fatty acid β-oxidation (FAO) for improved energy production, has also been shown to enhance T cell memory response [27]. Novel strategies to address these immunometabolic factors will improve our ability to generate effective immunotherapies.

## 2. Metabolic Pathways Influencing Antitumor Immune Function

### 2.1. Fuel Sources

T cells rely on multiple distinct metabolic pathways to meet their energy demands as they progress through differentiation, activation, and exhaustion. Typically, as T cells undergo proliferation and differentiation to effector T cells, they experience a shift towards glycolytic metabolism to fulfill their metabolic demands [28,29]. Most studies have shown that increased dependence on glycolysis is characteristic of short-lived effector T cells and is typically associated with exhaustion [22]. However, the exact implication of these findings in the setting of antitumor immunity is not entirely established, as several studies have also demonstrated that T cells exhibiting increased glycolysis can continue to exert effector and cytolytic function and maintain effective tumor control [30]. While early studies have established that the glucose transporter 1 (Glut1) is induced upon TCR stimulation and that the subsequently increased influx of glucose is essential for interferon-gamma (IFN-**γ**) production [31], later work has demonstrated that inhibiting glycolytic metabolism in T cells could also be advantageous in adoptive cell therapy (ACT) [22]. This study showed that promoting glycolytic flux drives CD8^+^ T cells toward a terminally differentiated state, while its inhibition preserves the formation of long-lived memory CD8^+^ T cells. In line with these findings, the inhibition of glycolysis via the reduced mammalian target of rapamycin (mTOR) signaling has been shown to shift T cell metabolism toward FAO and to promote the generation of memory T cells [32].

The roles of lipids as fuel sources and signaling molecules are also important in modulating the antitumor immune response. Memory T cells rely on the expression of the serine hydrolase enzyme, the lysosomal acid lipase (LAL), to utilize fatty acids for FAO to promote and sustain memory T cell development, linking cell-intrinsic lipolysis to metabolic reprogramming in lymphocytes and memory T cell fates [27]. A recent study also showed that lipid kinase acyl glycerol kinase (AGK) is vital for maintaining the metabolic fitness of CD8^+^ T cells, primarily by suppressing the phosphatase and tensin homolog (PTEN) and enhancing mTOR activity, thereby promoting antitumor activity [33]. Interestingly, our lab found that activated T cells express increased levels of sphingosine kinase-1 (SphK1), leading to enhanced levels of the intrinsic lipid sphingosine-1-phosphate (S1P) [34]. In the setting of antitumor immunity, the increased levels of S1P led to the activation of the lipid transcription factor peroxisome proliferator-activated receptor gamma (PPARγ), which enhanced regulatory T cell (Treg) development and limited antitumor activity [34]. Similarly, a recent study demonstrated that the expression of fatty acid-binding protein 5 (FABP5) plays a crucial role in maintaining mitochondrial integrity and modulates Treg function [35]. The inhibition of FABP5 in Tregs decreases OXPHOS and impairs lipid metabolism [35]. Thus, lipid metabolism and signaling play a diverse role in modulating the immune response, resulting in both pro-and anti-tumor effects. Additional work is needed to elucidate these multifaceted functions further.

In addition to the roles of glucose and fatty acids in shaping the T cell response mentioned above, recent studies have identified the nonessential amino acids serine and arginine as important metabolic sources that alter the antitumor response. Of note, an increase in L-arginine in activated T cells was found to be associated with a shift from glycolysis to OXPHOS [36]. These global metabolic changes were also associated with increased central memory T cells exhibiting superior antitumor activity in a murine tumor model [36]. Ma et al. demonstrated that even in glucose-rich environments, extracellular serine is necessary to support optimal T cell activation and proliferation [37]. Serine is processed via one-carbon metabolism upon T cell activation, supplying glycine and one-carbon units for nucleotide biosynthesis [37].

### 2.2. Oxidative Phosphorylation and Glycolysis

Many studies have identified increased the mitochondrial spare respiratory capacity (SRC) and enhanced OXPHOS as essential characteristics of memory T cells [26]. Specifically, van der Windt et al. demonstrated that memory T cells with enhanced SRC and OXPHOS upregulate mitochondrial fatty acid oxidation to support their metabolic demands [26]. Thus, a bulk of evidence suggests that a shift to mitochondrial oxidative metabolism in place of anaerobic glycolysis is associated with the development of memory T cells. Building on the importance of mitochondrial function in determining the differentiation state of T cells, Sukumar et al. demonstrated that the transfer of tumor epitope-reactive T cells with low mitochondrial membrane potential (ΔΨm) was associated with superior long-term in vivo persistence and an enhanced capacity to eradicate established tumors compared to the transfer of cells with high ΔΨm [38]. Furthermore, these same T cells differed in their ability to neutralize reactive oxygen species (ROS), which directly correlated to the differences in ΔΨm [38]. Given these findings, our lab demonstrated that T cells with low ΔΨm and low glycolysis could also be tracked using the expression of cell surface thiols (-SH), reflecting the antioxidant capacity of T cells [39]. Recently, the role of the critical antioxidant molecule glutathione (GSH) has also been implicated in regulating T cell function, where the ROS-dependent engagement of the metabolic signaling pathways was shown to reprogram inflammatory T cell responses [40]. Similarly, our group observed that employing recombinant thioredoxin (rTRx), another critical antioxidant molecule, programmed antitumor CD8+ T cells with high spare respiratory capacity and increased the persistence of T cells in vivo and led to enhanced tumor control upon ACT [41].

While the evidence presented above suggests that oxidative metabolism is favorable for the development of memory T cells, it must also be noted that increased glycolysis is not always correlated with the differentiation of short-lived effectors or a lack of robust tumor control. For example, in a study conducted by Doedens et al., the lack of the Von Hippel–Lindau (VHL) tumor suppressor molecule in mature T cells was shown to generate long-lived memory despite the impaired mitochondrial metabolism decreasing the spare respiratory capacity [42]. Furthermore, the VHL-deficient T cells displaying the constitutive activation of hypoxia-inducible factor 1-alpha (HIF1α) and enhanced constitutive glycolytic metabolism showed an equivalent ability to generate long-lived (>60 days) memory following acute viral infection [42]. Thus, contrary to previous studies suggesting that the mitochondrial metabolic pathways are uniformly essential for the generation of memory T cells, this study described memory formation in T cells with constitutive glycolytic metabolism, suggesting the fuel does not necessarily dictate function. Similarly, our group reported that a lack of p53 renders T cells as a highly glycolytic phenotype in 2016, and these p53^−/−^ CD8^+^ T cells exhibited high cytolytic activity with enhanced tumor control [43]. Notably, p53 is a known negative regulator of glycolysis [44], but VHL acts as a positive regulator of p53 [45].

### 2.3. Non-Metabolic Functions of Glycolysis-Associated Enzymes

Glycolytic pathway enzymes may also play roles in immune cell function outside of the classical functions within glycolysis. For example, the glyceraldehyde-3-phosphate dehydrogenase (GAPDH) is also a transcriptional repressor that binds to the 3′UTR of the IFNγ promoter [46]. Thus, reducing cytosolic GAPDH (increasing its involvement in glycolysis) resulted in higher IFNγ secretion upon glucose availability [46]. Conversely, another report showed that another glycolysis enzyme, lactate dehydrogenase A (LDHA), is induced in T cells upon activation to support aerobic glycolysis and promotes IFNγ expression independently of any interaction with the 3′UTR of the IFNγ promoter [47]. Similarly, the pyruvate kinase M2 (PKM2) isoform of the pyruvate kinase (PK), the glycolytic enzyme responsible for catalyzing the conversion of phosphoenolpyruvate to pyruvate, has been shown to play additional roles in the regulation of gene transcription and protein phosphorylation. For example, a recent study showed that the pharmacological activation of PKM2 in T cells using the allosteric activator TEPP-46 results in the significant inhibition of T cell activation, proliferation, and cytokine production, explicitly preventing the differentiation of Th17 and Th1 T cells [48]. Thus, in addition to the metabolites produced in the T cell metabolic pathways, the enzymes themselves may play essential roles in regulating the critical genes involved in T cell activation and function.

## 3. Alterations of Metabolic Pathways in the Tumor Microenvironment

Differences in metabolic pathways appear to play a vital role in the context of the tumor microenvironment (TME). In most solid tumors, the TME involves a distinct set of metabolic factors that favor tumor growth and that inhibit antitumor immune function. Recent studies have demonstrated that tumor-infiltrating lymphocytes (TILs) become exhausted and dysfunctional within the TME and that this is partially due to deficits in glycolytic and oxidative metabolism [24,31,49].

### 3.1. Hypoxia in the TME

Due to the unrestricted proliferation of cancer cells, which tends to exceed the vascular perfusion of the TME, most tumors have a hypoxic environment [50]. In response to this hypoxia, cancer cells upregulate hypoxia-inducible factor 1-alpha (HIF1α), which causes increased glucose uptake and glycolytic metabolism and a resultant increase in lactate release into the TME [50]. Importantly, this lactate production impairs T cell activation and function and especially blunts the activation of NFAT and the production of IFNγ [49,51]. The elevated expression of LDHA, the primary enzyme that is responsible for producing lactate, is associated with poorer outcomes in cancer patients [49]. In a murine model, reductions in lactic acid production (LHDA^low^) resulted in slower tumor growth with the increased infiltration of IFNγ-producing T cells in the tumors, suggesting that LDHA may be an important therapeutic target for improving immunotherapies [49].

The hypoxic nature of the TME also has a direct impact on TILs and has both suppressive and stimulatory effects on T cells [52]. For example, it has been demonstrated that increased HIF1a activity is associated with enhanced glycolysis, migration, and cytotoxic effector function in CD8^+^ T cells [42,53]. In addition, the inhibition of the von Hippel–Lindau (VHL) tumor suppressor, the primary negative regulator of HIF1a, results in CD8^+^ T cells showing an enhanced ability to control cancer growth [42]. These results suggest that the hypoxic nature of the TME and the subsequent upregulation of HIF1α expression would result in enhanced antitumor T cells function; however, other evidence suggests that the hypoxic TME is overall suppressive to TILs [54,55]. Notably, multiple studies have demonstrated that the hypoxia-induced expression of HIF1α in tumor cells directly upregulates the expression of programmed death-ligand 2 (PD-L1 and PD-L2) on tumor cells [56,57,58]. The interaction of these ligands with PD-1 expression on T cells within the TME is a critical mechanism by which tumor cells suppress the antitumor immune response and induce T cell exhaustion [56,57].

Interestingly, strategies to decrease hypoxia within the TME have shown some efficacy in improving immunotherapies [59]. For example, Hatfield et al. demonstrated that in multiple murine cancer models, the superoxide significantly reverses intra-tumoral hypoxia and extracellular adenosine levels, resulting in enhanced TIL infiltration and increased activity in terms of pro-inflammatory cytokine production, reduced immunosuppressive signaling molecules, and reduced tumor growth [59]. Sharping et al. achieved similar results by combing metformin adjuvant therapy with PD-1 blockade therapy [60].

### 3.2. Immunosuppressive Metabolites in the TME

In addition to hypoxia and elevated lactic acid, the TME is also characterized by significantly elevated levels of the tryptophan-derived catabolite kynurenine. Kynurenine is produced by the enzyme indoleamine 2,3-dioxygenase (IDO), which is typically highly expressed by tumor cells as well as in tumor-associated macrophages (TAMs) and myeloid-derived suppressor cells (MDSCs) in the TME [61,62,63]. Higher IDO expression levels are associated with poorer outcomes in patients with gastric adenocarcinoma [64]. Kynurenine has been shown to inhibit the proliferation and effector function of effector T cells and to induce the expansion of Tregs [61,65]. Conversely, the inhibition of IDO can promote the conversion of Tregs to proinflammatory Th17 cells [66]. In addition to kynurenine production, IDO disrupts T cell function in the TME through the depletion of tryptophan, essential nutrition for effector T cells [67]. Depleted tryptophan levels are sensed by the general control nonderepressible 2 (GCN2) kinase, a stress response kinase that induces a decrease in global protein synthesis and that ultimately leads to T cell anergy in CD8^+^ T cells [67]. These discoveries have led to the development of IDO inhibitors as “immunometabolic adjuvants” for cancer therapy, several of which are currently being evaluated in clinical trials [68,69].

TAMs and Tregs also produce high levels of the immunosuppressive metabolite adenosine within the TME [70]. Both Tregs and TAMs express CD39 and CD73, which are the surface ectonucleotidases that are responsible for converting ATP to adenosine. The adenosine that is produced interacts with the A2a receptor and acts as an immunosuppressant to T cells, inhibiting the TCR signaling and expression of the IL-2 receptor while also upregulating the expression of immune checkpoint molecules [70]. In addition, adenosine signaling has also been shown to impair the metabolic fitness of CD8^+^ TILs by impairing both oxidative phosphorylation and glycolysis in an A2a receptor-dependent manner [71]. These findings have led to several pharmacological inhibitors and monoclonal antibodies targeting CD39, CD73, and the adenosine A2a receptor, which are currently being evaluated in clinical trials [70].

### 3.3. Metabolic Competition in the TME

Several studies have identified metabolic competition for nutrients in the TME as a significant contributor to impaired antitumor T cell function [30,72,73]. For example, in a mouse sarcoma model, it was demonstrated that the highly glycolytic nature of T cells depletes intratumoral glucose levels, metabolically restricting T cells and leading to dampened mTOR activity glycolytic capacity and IFNγ production [30]. In this model, immune checkpoint blockade therapy resulted in the elevation of intratumoral glucose levels and the restoration of antitumor T cell function [30]. While these results suggest that competition for glucose between cancer cells and T cells within the TME contributes to T cell dysfunction, recent work from Reinfeld et al. alternatively suggests that glucose is not broadly limited in the TME and that instead, cell-intrinsic programs drive the preferential acquisition of glucose, glutamine, and lipids by the different cell types in the TME [74]. This study found that human renal cell carcinoma and mouse subcutaneous MC38 tumor samples had comparable levels of glucose and glutamine when matched to healthy tissue [74]. Across a range of cancer models, the authors found that myeloid cells had the most significant capacity to take up intratumoral glucose, followed by T cells and cancer cells. In contrast, cancer cells had the highest ability to consume glutamine and lipids. Interestingly, this cell-intrinsic nutrient partitioning was partly dictated by the mTORC1-driven uptake of glutamine, suppressing the expression of glycolysis-related genes. It was found that restricting glutamine uptake enhanced glucose uptake and glycolytic activity in TILs [74]. It must be noted that nutrient availability within tumors is likely a dynamic state that depends on tumor type and stage; thus, further studies to investigate the concepts of nutrient competition and cell-intrinsic programs that dictate metabolite utilization in the TME are warranted.

### 3.4. Organoid Methods for Studying the TME

Complex and dynamic cellular interactions govern the TME. To test and monitor the efficacy of immunotherapies, increasingly robust models are needed; the newly developed three-dimensional organoid culture methods allow for the greater incorporation of immune components [75,76,77]. Organoid methods can now propagate human tumor biopsies in vitro, allowing for the establishment of large tumor biobanks and future approaches for personalized medicine [75]. Zhang et al. engineered mouse fallopian tube epithelial organoids to generate the multiple mutational combinations seen in high-grade carcinomas, for which they revealed genotype-dependent similarities in terms of chemosensitivity, secretome, and immune microenvironment [78]. The continued development of this methodology can expect exciting advances and translational applications.

### 3.5. Strategies to Restore Metabolic Pathways

The restoration of these metabolic pathways in T cells within the TME, for example, by increasing the production of the glycolytic intermediate phosphoenolpyruvate, has been shown to improve their antitumor function and ability to persist within the TME [23]. In a recent study conducted by Gemta et al., ENOLASE-1 was identified as a downregulated target enzyme in TILs within the TME, resulting in repressed glycolytic activity and impaired effector function [24]. This function could be restored by supplementation pyruvate (a downstream product of ENOLASE-1) to bypass the inhibition of ENOLASE-1 observed in the TME [24]. Similarly, in another study, the overexpression of peroxisome proliferator-activated receptor gamma coactivator 1-alpha (PGC1α, a master regulator of mitochondrial biogenesis) on TILs acted to bolster their OXPHOS capacity and to enhance their anti-tumor function [79,80]. Factors within the TME act to disrupt metabolic pathways within T cells to promote the immune escape of tumor cells, and further investigation should be conducted regarding potential strategies to overcome these disruptions.

**Figure 1:**
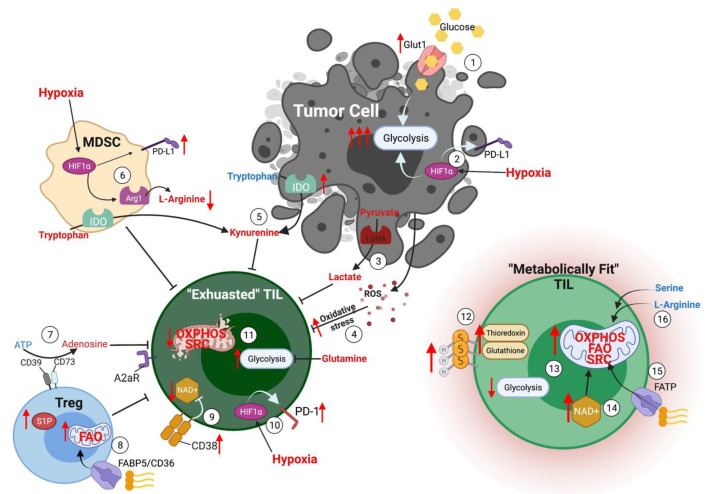
Metabolic conditions in the TME. (1) Tumor cells express high levels of glucose transporters to drive glycolytic metabolism. (2) The hypoxic nature of the TME promotes HIF1α expression *in tumor cells*, resulting in upregulation of glycolytic metabolism and PD-L1 expression. (3) High concentrations of lactate in the TME secondary to tumor cell glycolysis result in T cell suppression. (4) Elevated ROS in the TME induces oxidative stress in T cells. (5) Both tumor cells and MDSCs express high levels of the enzyme IDO, consuming tryptophan and producing high levels of kynurenine, which acts as an immunosuppressive metabolite. (6) Hypoxia drives HIF1α expression in MDSCs, promoting the expression of PD-L1 and the enzymes arginase-1, which reduces the availability of L-arginine in the TME. (7) CD39 and CD73 expressed on Tregs convert ATP to the immunosuppressive metabolite adenosine, which binds to the A2aR receptor on effector T cells. (8) Tregs express high levels of fatty acid transporters to support mitochondrial fatty acid oxidation, enhancing their ability to exert their immunosuppressive function on effector T cells. (9) Exhausted TILs express high levels of CD38, which acts to deplete NAD^+^, a metabolite required for optimal T cell function. (10) Hypoxia drives HIF1α expression in T cells, resulting in the upregulation of the immune checkpoint molecule PD-1. (11) Exhausted TILs rely heavily upon glycolytic metabolism and have impaired mitochondrial oxidative phosphorylation and spare respiratory capacity. (12) Metabolically fit TILs express high surface thiols and key antioxidant molecules, including thioredoxin and glutathione. (13) Metabolically fit TILs are characterized by high spare respiratory capacity and enhanced ability to use oxidative phosphorylation and fatty acid oxidation to support their effector functions. (14) Elevated levels of NAD+ support the activity of Sirt1 and contributes to post-translational modifications and epigenetic stability, resulting in metabolically fit T cells. (15) Expression of fatty acid transporters on T cells supports their ability to utilize fatty acids as a fuel source via fatty acid oxidation. (16) Serine and L-arginine are important metabolites for effector T cells to support the antitumor T cell response.

One such strategy that was demonstrated by Rivadeneira et al. involves using oncolytic viruses to metabolically reprogram TILs within the TME [81]. In this study, oncolytic viruses were engineered to express leptin in tumor cells. Leptin has been identified as a potent metabolic reprogramming agent, and melanoma cells express leptin-induced superior metabolic function and memory phenotype in T cells within the TME [81]. These metabolically superior TILs induced complete response in tumor-bearing mice with persistent antitumor memory [81]. Another study identified immune checkpoint blockade therapy (using antibodies again CTLA-4, PD-1, and PD-L1) as a strategy to increase glucose in the TME, thereby supporting T cell glycolysis and effector function [30]. At the same time, a PD-L1 blockade on tumor cells was shown to inhibit tumor cell glycolysis by inhibiting mTOR signaling and downregulating glycolysis enzymes, further contributing to superior tumor control [30]. To overcome nutrient competition within the TME, Qiu et al. demonstrated that the treatment of glucose-restricted CD8^+^ T cells with metabolite acetate rescues their effector function [82]. This study showed that acetate promotes histone acetylation and chromatin accessibility and enhances IFNγ gene transcription and cytokine production in an acetyl-CoA synthetase (ACSS)-dependent manner. While ex vivo acetate treatment increased IFNγ production by exhausted T cells, reducing ACSS expression in T cells impaired IFNγ production by TILs and prevented tumor control [82]. Thus, this study demonstrated that hyporesponsive T cells could be epigenetically remodeled and reactivated by acetate, suggesting that the pathways regulating the use of substrates alternative to glucose could be therapeutically targeted to promote T cell function during cancer. A summary of the metabolic conditions within the TME is graphically depicted in Figure 1.

## 4. Metabolic Preferences of T Cell Subsets

### 4.1. Th17 T Cells

Several studies have now established that both cytolytic CD8^+^ T (Tc) cells and CD4^+^ T helper (Th) cells are effective in tumor immunotherapy [39,83,84,85,86,87]. Lately, intensive investigation has been focused on Th17 cells, which effectively control tumor growth in an interferon-gamma (IFNγ)-dependent manner and exhibit increased persistence due to their “stem cell-like” phenotype [88,89,90]. However, the ex vivo programming of Th17 cells in the presence of TGFβ increases the cell surface expression of ectonucleotidases CD39 and CD73, increasing susceptibility to immunosuppression and reducing effector functions [87]. Our lab has previously shown that the ATP-mediated suppression of IFNγ production by Th17 cells can be overcome by either the genetic ablation of CD73 or by generating TGFβ-independent Th17 cells in the presence of IL-1β [87]. Th17 cells cultured in IL-1β are also highly polyfunctional, express high levels of effector molecules, and exhibit better short-term control of B16-F10 murine melanoma despite reduced stem cell-like properties [87,91]. Importantly, we deciphered that adding TGFβ at a low dose that does not up-regulate CD73 expression but that still induces stemness drastically improves the antitumor function of IL-1β cultured Th17 cells [87]. The effector property of IL-1β-dependent Th17 cells is due to their high glycolytic capacity since generating IL-1β-dependent Th17 cells in pyruvate-containing media impairs glycolysis as well as its anti-tumor potential [87]. Along similar lines, a recent study showed that the administration of IL-1β increased the population size and functionality of adoptively transferred T cells within the tumor microenvironment, which is primarily mediated by IL-1β-stimulated host cells [91]. These studies also underscore the potential of provoking inflammation within the tumor microenvironment, which modulates cellular metabolism to enhance antitumor immunity. Another recent research focusing on the Th17 subset found that mitochondrial OXPHOS serves to dictate the fate decision of T cells toward the Th17 subset, as the upregulation of OXPHOS promotes the expression of the basic leucine zipper ATF-like transcription factor (BATF, a critical transcription factor for Th17 differentiation) and also promotes TCR and mTOR signaling [92].

### 4.2. Th1/17 Hybrid T Cells

To achieve robust tumor control via adoptively transferred T cells, our lab generated a hybrid Th1/17 (and Tc1/17) subset using novel ex vivo programming conditions that led to an increased proportion of T cells co-secreting high levels of IFNγ (Th1 signature cytokine) and IL-17 (Th17 signature cytokine) [93]. These hybrid cells could control tumor growth in mice for more than 120 days [93]. This endeavor supported our hypothesis that bringing together the antitumor effector function of Th1 cells and the stemness properties of Th17 cells could lead to robust tumor control due to the persistence of long-lived antitumor T cells that maintain an effector phenotype. Given the powerful tumor control exhibited by ex vivo programmed Th1/17 cells, we further identified the molecular and metabolic signatures of these cells. Comprehensive metabolite analysis revealed the enhanced accumulation of the vital metabolite nicotinamide adenine dinucleotide (NAD^+^) [93]. Notably, the ectonucleotidase CD38, an NAD^+^ hydrolase that is inversely correlated to NAD^+^ levels, was decreased in hybrid Th1/17 cells [94,95]. Since NAD^+^ is a crucial substrate for the histone deacetylase Sirt1, we observed increased protein deacetylation and a unique metabolic profile in hybrid Th1/17 cells [93,96]. Metabolically, hybrid Th1/17 cells uniquely exhibit increased glutaminolysis and an increased dependence on the mitochondrial metabolism [93]. The role of glutamine metabolism in determining stem cell fate and Treg/Th17 balance has been shown previously [97,98]. Similarly, Sirt1 can also regulate methyltransferase activity and influence methylation [99,100]. Thus, the robust antitumor function of hybrid Th1/17 cells is likely mediated by an overall rejuvenated T cell phenotype due to high NAD^+^, which influences a combination of events, including post-translational modifications and epigenetic stability, leading to a metabolically fit antitumor memory T cells.

A recent study demonstrated that T cell plasticity and metabolic differences could be noted in vivo [101]. This study showed that Th17 cells in a mouse model of autoimmune disease are functionally and metabolically heterogeneous [101]. While one subset contained stemness-associated features but lower anabolic metabolism, another subset exhibited a reciprocal phenotype with a higher metabolic activity supporting trans-differentiation into Th1-like cells [101]. Thus, heterogeneous populations of T cells display differences in terms of metabolic commitment, even within classically defined T cell subsets. Strategies that can uniformly reprogram patient-derived T cells will be necessary for achieving reproducible results with immunotherapy.

### 4.3. IL-9-Secreting T Cells

Another T cell subset that has gained recent attention is one that secretes high levels of IL-9. Both CD4^+^ Th9 and CD8^+^ Tc9 cells exhibit a superior ability to control tumor growth compared to their Th1 or Th17 counterparts [102]. A critical difference in Th9 cells is their ability to secrete IL-10 and express the transcription factor signal transducer and activator of transcription 6 (STAT6) and GATA-3 [103]. Pegylated IL-10 has been previously shown to improve tumor control [104]. GATA-3, a Th2 signature transcription factor, is also important for maintaining a stem cell-like phenotype [105]. This is in line with previous observations that Th2 clones are less susceptible to cell death than Th1 clones [106]. The effector functions of Th9 cells are more dependent on glycolytic activity; thus, Sirt1 activity may not be necessary for these cells; however, it remains to be seen how mitochondrial fitness is maintained in the absence of the deacetylation of PGC1α, which is essential for mitochondrial biogenesis [107,108].

### 4.4. Regulatory T Cells

Another essential T cell subset includes regulatory T (Treg) cells. Tregs are responsible for peripheral immune tolerance, maintaining tolerance to self-antigens and preventing autoimmunity [109]. Tregs are defined by the lineage-defining expression of the transcription factor Foxp3, which is essential for their differentiation and immune-suppressive function [109]. While Tregs protect the host by limiting an excessive immune response in autoimmunity, in cancer settings, their immunosuppressive function blunts the effector T cell response against tumor cells and contributes to tumor growth, negatively impacting host survival [110]. Treg stability is reliant on a unique metabolic profile. Several studies have indicated that Treg cells are less dependent on glycolysis and instead rely primarily on OXPHOS to fulfill their energy needs and suppressive activity [111,112]. Compared to effector T cell subsets, Tregs exhibit increased mitochondrial mass to facilitate OXPHOS [113,114]. Likewise, reports have also revealed that increased glycolysis is correlated to reduced Treg induction and stability [115]. The deletion or inhibition of signaling molecules that promote glycolysis, such as transcription factor HIF-1α, leads to an increase in Foxp3 induction and enhanced Treg stability [115]. This unique metabolic preference of Tregs endows them with a survival advantage in the TME. As has been well established, tumor cells undergo a metabolic shift from OXPHOS to aerobic glycolysis, leading to the consumption of environmental glucose and glutamine [116], and the depletion of these critical nutrients in the TME renders conventional T cells unresponsive or functionally exhausted [113,117]. Additionally, glucose deprivation primes T cell differentiation from conventional T cells towards Tregs by promoting Foxp3 expression [116,118,119]. The depletion of specific amino acid transporters (notably ASCT2 and SLC7a5) has been shown to have minimal impact on Treg differentiation. In contrast, amino acids (especially glutamine and leucine) are essential in promoting T cell differentiation into Th1, Th2, and Th17 subsets via the activation of mTORC1 [120,121]. Thus, these amino acids appear to preferentially drive effector T cell rather than Treg differentiation [120,121,122]. Depleting glutamine from the culture medium has enhanced the differentiation of naïve CD4^+^ T cells into Treg cells even in the presence of Th1 promoting cytokines [118]. This is likely due to the decreased intracellular concentrations of the metabolite α-ketoglutarate (α-KG), which is found when extracellular glutamine is limited [118]. These findings illustrate how glucose and glutamine deprivation within the TME promotes Treg generation and shifts the balance of the immune response to become more suppressive.

The role of fatty acids and FAO in regulating Treg function and stability has also been explored in multiple studies. In mouse models, Tregs have been shown to preferentially uptake fatty acids and undergo FAO to support their metabolism, while short-chain fatty acids simultaneously help Treg differentiation [119]. Tregs are metabolically programmed to increase SRC and OXPHOS activity. This enhanced mitochondrial activity is fueled by a much greater uptake of long-chain fatty acids than conventional T cells [111]. Another study demonstrated that Tregs reserve fatty acids in lipid droplets in the form of di- and triglycerides and phospholipids. These lipid droplets play an important role in fuel storage, protecting cells from lipotoxicity and reducing protein kinase C activity to drive Foxp3 expression [119]; ultimately Tregs rely on a combination of glycolysis, fatty acid synthesis, and FAO for their survival and proliferation in the glucose-restricted TME, allowing Tregs to prevail over conventional T cells, which primarily rely on the glycolytic metabolism of glucose to meet energy demands [123]. These data suggest that Foxp3 expression promotes a metabolic state that enhances its stability and that protects the cells in environments with reduced glucose and elevated fatty acids, such as in the TME, at the same time. Understanding these metabolic preferences of the Tregs within the TME has paved the way for several potential therapeutic strategies. For example, Tregs that express high levels of CD36 and SLC27A1 (fatty acid transporters) in murine brain tumors can be targeted by restricting lipid uptake with sulfo-N-succinimidyl oleate (SSO) or FAO with etomoxir, ultimately limiting the immunosuppressive capacities of the Tregs and prolongs survival [124].

Similarly, a recent article revealed that the Tregs residing in the TME upregulate CD36 expression to modulate mitochondrial biogenesis and NAD^+^ levels, supporting their survival and functional fitness through a peroxisome proliferator-activated receptor-β (PPAR-β)-dependent mechanism [125]. The additive antitumor effects triggered by combined treatment with PD-1 and CD36 blockade provide broad therapeutic potential without disrupting immune homeostasis in patients with cancer [125]. A more detailed investigation is needed to determine the essential nutrients, metabolites, and metabolic pathways that influence different T cell subset differentiation, proliferation, and function in different tumor settings. This information will ultimately provide potential novel targets for immunotherapy in cancer. An updated summary of current metabolites under investigation and their potential role in antitumor immunity is represented in Table 1.

## 5. Influence of Co-Stimulation on T Cell Metabolism

The metabolic state of T cells is a dynamic process that changes with the different stages and activation states of T cells [131]. Upon optimal T cell activation, in which TCR stimulation is accompanied by proper co-stimulation, naïve T cells not only expand in size but also exhibit enhanced expression of glucose transporters (i.e., Glut1) that aid in glucose uptake and fuel glycolysis, which is required to support their growth, proliferation, and effector functions [132]. Notably, T cells that are *immunologically* anergic due to lack of proper co-stimulation by CD28 engagement have also been shown to be *metabolically* anergic [133]. A study conducted by Zheng et al. showed that one mechanism that is responsible for the maintenance of T cell immunological anergy is a failure to up-regulate the specific metabolic machinery required to support the increased metabolic requirements of T cell activation [133]. Emerging data also demonstrates that CD28 costimulation activates the initial steps of both glycolysis and the mTOR cascade, a signaling pathway that regulates a variety of crucial metabolic machinery including nutrient transporters [28,134,135,136]. Co-stimulation by CD28 has been shown to upregulate the expression of both Glut1 (the primary importer of glucose) and hexokinase 2 (the enzyme involved in the first step of glycolysis) [119].

The co-stimulation of T cells with CD28 imprints mitochondria with a latent metabolic capacity, which is essential in shaping the memory T cell response [16]. The early engagement of T cell mitochondria in a CD28-dependent fashion was shown to contribute to a robust T cell memory phenotype, as indicated by remodeling of cristae, enhancement of spare respiratory capacity (SRC), and rapid cytokine production upon production restimulation [16]. Similar studies have demonstrated the role of the secondary co-stimulatory molecule 4-1BB (CD137) in enhancing mitochondrial biogenesis and in improving tumor control when synergistically used with PD-1 blockade (a co-inhibitory molecule) [17]. In line with these data, the inclusion of 4-1BB in the CAR architecture promotes the expansion of CD8^+^ central memory T cells with enhanced respiratory capacity, fatty acid oxidation, and mitochondrial biogenesis [137]. Furthermore, as demonstrated by Kawelekar et al., 4-1BB CAR T cells increased in vivo persistence compared to CAR T cells constructed with CD28 domains and primarily had an effector memory phenotype and were heavily utilized during glycolytic metabolism [137]. These findings are corroborated by another study showing that 4-1BB co-stimulation reduces the T cell exhaustion induced by CAR signaling more effectively than CD28 co-stimulation [138].

ICOS (Inducible T-cell Co-Stimulator), a co-stimulatory molecule belonging to the CD28 superfamily, has been shown to activate mTOR to upregulate glucose uptake and metabolism upon ligation [139]. Furthermore, the inclusion of ICOS in a CAR construct and 4-1BB has been shown to increase CAR T cell persistence and efficacy in solid tumor models [140]. GITR, another costimulatory receptor in the same superfamily as 4-1BB, has also been recently studied as a potential target for improving cancer immunotherapy due to its role in altering T cell metabolism [141]. The Agonism of GITR on CD8^+^ T cells has been shown to increase the oxygen consumption rate, basal glycolysis, and glycolytic capacity in a mouse tumor model [141]. Thus, the role of costimulatory signaling molecules in regulating T cell metabolism and mitochondrial function is beginning to be revealed and will likely be important in the design of future immunotherapies.

In addition to the costimulatory molecules described above, T cells express several coinhibitory receptors that ultimately influence the cell’s metabolic state. The coinhibitory receptor PD-1 has been linked to glycolytic capacity and mitochondrial function [139]. Multiple groups have shown that blocking PD-1 enhances glycolytic ability and mitochondrial function in both virus-specific CD8^+^ T cells and TILs [80,139,142]. Thus, while proper costimulation appears to enhance T cell metabolism, the engagement of coinhibitory receptors appears to induce an impaired metabolic state that renders T cells anergic.

## 6. Cytokine Signaling in Dictating T Cell Metabolism

Growth factors and cytokines are critically important for sustaining activated T cells in vivo and for regulating the balance between activation and tolerance [143,144,145,146]. While IL-2, IL-7, IL-15, and IL-21 have been shown to boost the T cell immune response and to promote T cell effector function, IL-10 and TGFβ typically function to keep activated T cells in check and to rein in the immune response [146,147]. Early clinical trials attempting to engage antitumor T cells to control malignancies involved the use of high doses of IL-2 injected systemically [148,149,150,151,152,153,154]. The T cells that were generated in these conditions were called lymphokine-activated T cells [148,150,155]. While this strategy received mild success, it failed to demonstrate a long-term antitumor effect and had significant toxicity [152,156,157,158]. In light of our understanding of the differential role of IL-2 and IL-15, we now know that IL-2 induced T cells with a high effector phenotype would have been highly glycolytic and categorized as short-lived effector T cells rather than more persistent memory T cells [26,159,160,161].

### 6.1. IL-15

IL-7 and IL-15, which are members of the common gamma-chain family of cytokines, are considered powerful pro-inflammatory cytokines and can destabilize chromosomes and induce tumorigenesis [162]. However, IL-7 and IL-15 have been shown to induce human memory stem T cells from naïve precursors [163]. IL-15 has been shown to enhance antitumor immunity, and a recent next-generation IL-15 that is activated inside the TME has recently been shown to selectively enhance the stem-like properties and antitumor efficacy of intratumoral T cells and NK cells while limiting systemic toxicity [164]. Several reports have demonstrated that, metabolically, IL-2 is a cytokine that enhances glycolysis in T cells [27], while IL-15 more effectively upregulates T cell mitochondrial respiration by promoting the expression of the lysosomal hydrolase LAL (lysosomal acid lipase) to mobilize fatty acids for FAO through cell-intrinsic lipolysis [27]. Our lab has recently shown that conventional TCR activation leads to the increased expression of the sphingosine kinase-1 (SphK1) and enhanced S1P levels, which hamper lipolysis and mitochondrial respiration [34]. However, the memory T cells generated with IL-15 treatment maintain low levels of Sphk1, promoting cell-intrinsic lipolysis [34]. Another study showed that IL-2-treated effector T cells have punctate mitochondria, whereas the IL-15-generated memory T cells exhibit a fused mitochondrial network [165]. An improvement in antitumor T cell response was observed when effector T cells were engineered with a fusion phenotype by regulating Opa1 expression or by exposing the cells to the mitochondrial fusion promoter M1 and the mitochondrial fission inhibitor Mdivi-1 [165]. Mitochondrial fusion in T cells configures electron transport chain (ETC) complex associations that favor OXPHOS and FAO along with compact cristae. In contrast, fission in T cells leads to cristae expansion, reducing ETC efficiency and promoting aerobic glycolysis [165]. Thus, mitochondrial remodeling appears to be a critical signaling mechanism that instructs T cell metabolic programming and that regulates IL-2 and IL-15 signaling dynamically.

### 6.2. IL-7

IL-7 is an essential nonredundant cytokine required for T cell development and survival with a unique impact on T cell metabolism [166,167,168,169,170,171]. The role of IL-7 in promoting Glut1 trafficking and glucose uptake via the STAT5-mediated activation of Akt to support T cell survival has been demonstrated in vitro [172]. Additionally, in vivo studies using IL-7 receptor-conditional deletion showed that IL-7 receptor deficiency leads to T cell atrophy, which is characterized by delayed mitogenesis and reduced glycolytic flux [173]. It has also been shown that engineering antitumor T cells with a constitutively active IL-7 receptor results in durable tumor regression [174]. Triglyceride synthesis is a central component for the IL-7-mediated survival of human and mouse memory CD8^+^ T cells and depends on the expression of the glycerol channel aquaporin 9 (AQP9) [175].

### 6.3. IL-12

IL-12, one of the critical cytokines known to promote the Th1 subset of T cells, has also improved T cell-mediated tumor control in pre-clinical models [176]. It enhances the retroviral transduction efficiency of TCRs while preserving the effector function and expansion potential of the transduced T cells [177]. CD8^+^ T cells activated with exogenous IL-12 have elevated IL-7 receptor expression and rely on IL-7 for persistence and antitumor immunity [178]. The pre-treatment of T cells with IL-12 in vitro enhances the release of a range of cytokines (including IFNγ, TNFα, IL-13, IL-4, and IL-10), potentially by altering certain TCR signaling pathways (including increased pAkt, p-p38, and p-Lck signaling) and by enhancing oxidative metabolism [179]. In the setting of viral immunity, the IL-12 treatment of exhausted (PD-1^hi^) virus-specific CD8^+^ T cells reinvigorates mitochondrial activity and rescues IFNγ production, even in the absence of glucose [180].

### 6.4. IL-21

IL-21 is another pleiotropic cytokine that has unique effects on T cells by promoting effector function while reducing effector differentiation [181]. Notably, IL-21 treatment has been shown to increase T cell persistence and the stem cell memory phenotype and to improve tumor control in mouse models and patients [182,183]. Metabolically, IL-21 shifts the T cell metabolic phenotype from aerobic glycolysis towards fatty acid oxidation (FAO) [184].

### 6.5. IL-10

IL-10, conventionally considered an anti-inflammatory cytokine, has recently been identified as an important cytokine that regulates the cellular metabolism and exhaustion status of antitumor CD8^+^ T cells [185]. In multiple murine tumor models, treatment with the peritumoral administration of half-life-extended interleukin-10–Fc fusion protein promoted the expansion of terminally exhausted CD8^+^ TILs and promoted the effector function and enhancing oxidative phosphorylation in these exhausted T cells [185]. These findings suggest that IL-10 may play an important role in metabolically reprogramming terminally exhausted CD8^+^ TILs to upregulate mitochondrial pyruvate carrier-dependent oxidative phosphorylation and enhance antitumor capacity [185]. A summary of the recent preclinical work performed using animal models demonstrating the efficacy of targeting cytokine signaling and cellular metabolism is presented in Table 2. Clinical trials targeting IL-7, IL-12, IL-15, and IL-21 cytokine signaling in immunotherapy are ongoing; outcomes and study status are reported in Table 3.

## 7. Epigenetics

DNA methylation, histone post-translational modifications, and chromatin remodeling represent extensively studied epigenetic mechanisms that influence chromatin structure and gene expression without altering DNA sequences and that ultimately regulate a wide variety of biological processes. Recent studies from tumor and infectious disease models have revealed that various epigenetic mechanisms play a role in regulating effector and memory T cell function and metabolism [208,209,210,211,212]. For example, a pioneering study from Phil Greenberg’s group showed that the failure of inhibitory checkpoint PD-1 blockade to fully restore effector functions and reverse exhaustion of day 35+ TILs was attributable to the unchanged high expression of numerous other inhibitory receptors (such as TIM3 and LAG3). Notably, the exhausted TILs increased the molecule expression that is involved in epigenetic modifications and the induction of a repressive chromatin state, leading to irreversible T cell dysfunction [211]. Thus, strategies to overcome this dysfunction mediated by antigen stimulation and cell-intrinsic epigenetic modification are required to improve the outcome of ACT [212,213,214].

The association between cellular metabolism and epigenetic changes has been intensively studied in cancer cells; however, how these two critical biological pathways interplay to modulate T cell differentiation and function in the context of tumors is a relatively young field that has recently been attracting. The ability to modify T cell function through interventions that target vital metabolites or pharmacological agents that target the epigenome holds significant potential to improve current cancer immunotherapies.

### 7.1. Histone Acetylation

Histone post-translational modifications through acetylation and the deacetylation of lysine residues are the best-studied epigenetic programs. The acetylation of lysine residues is catalyzed by histone/protein acetylase (HAT) enzymes and reversed by histone/protein deacetylase (HDAC) enzymes [215]. HATs neutralize the otherwise positive charge of a lysine’s ε-amino group, allowing for increased the accessibility of the DNA for transcription or transcription factors. Conversely, deacetylation by HDACs removes acetyl groups from acetylated histones, leading to chromatin condensation and a repressive chromatin structure [215]. Acetyl-CoA (primarily produced in the mitochondrial matrix) is the central metabolite transferred by HATs for histone acetylation [216]. Thus acetyl-CoA acts as a crucial signal transducer that regulates the expression of a variety of genes and distinct cell differentiation programs influencing the acetylation status of histones. It has been reported that the acetylation of the transcription factor Foxp3 improves its ability to bind DNA and confers suppressive functions to Tregs [217]. Further, inhibition of the HAT p300 reduces Foxp3 acetylation, hampering Treg suppressive function and iTreg formation and promoting anti-tumor immunity [218]. Sirtuins (SIRTs) are one of the most prominent HDAC isoforms and have been intensively studied in the context of T cells. SIRTs require NAD^+^ for their deacetylation function [126]. Increased glycolytic activity and the decreased expression of the NAD^+^-dependent deacetylase SIRT1 have been linked to the proteasomal degradation of the forkhead box protein (FoxO1) in a population of terminally differentiated T cells [127].

### 7.2. Histone Methylation

Unlike histone acetylation and deacetylation, histone methylation displays a more complex and context-dependent relationship with chromatin states [219,220]. For example, while the tri-methylation of lysine 4 on histone 3 (H3K4me3) triggers transcription, tri-methylation of histone 3 on lysine 27 (H3K27me3) is associated with gene silencing. Importantly, histone methyltransferases use S-adenosyl-methionine (SAM) as their donor for methyl groups [221]. Thus, the SAM, which is produced from methionine via one-carbon metabolism, plays a crucial role in T cell proliferation and differentiation. It has been reported that antigen receptor engagement controls flux through the methionine cycle and RNA and histone methylations, which is dependent upon methionine transporter expression [222]. Methionine is required for maintaining intracellular SAM pools in T cells, as methionine restriction reduces histone H3K4 methylation (H3K4me3) at the promoter regions of the essential genes involved in Th17 cell proliferation and cytokine production [223]. Unlike histone methylation, which is linked to both gene transcription and silencing, an investigation conducted by Youngblood et al. reported that de novo DNA methylation is a critical mechanism for establishing T cell exhaustion that is acquired during a chronic viral infection or tumor challenge [224]. The data also identified Dnmt3a-mediated de novo DNA methylation as a major obstacle limiting the efficacy of PD-1 blockade therapy; thus, reversing these methylation programs has broad implications for novel approaches to increase T cell-based immunotherapies [224]. Several independent research groups have identified the transcription factor thymic selection-associated high mobility group box protein (TOX) as a critical indicator of T cell exhaustion and an initiator of the T cell exhaustion-specific epigenetic program. Based on these studies, the expression of TOX and TOX-dependent epigenetic programming appears to be essential for the high expression of inhibitory receptors and for maintaining different chromatin accessibility states in effector, memory, and exhausted T cells [225,226,227,228,229].

DNA methylation has also been implicated in maintaining T cell memory, specifically in the setting of antitumor immunity [230]. He et al. identified Ezh2, a histone methyltransferase, as a critical regulator of CD8^+^ T memory precursor formation [230]. Precisely, the methyltransferase activity of Ezh2 activates Id3 and silences Id2, Prdm1, and Eomes to promote the differentiation of memory precursor cells into functional memory cells [230]. This study builds on several previous studies that have demonstrated the role of histone methylation in regulating gene transcription patterns to influence the fate and function of both CD4^+^ and CD8^+^ T cells [231,232,233]. H3K27me3 and H3K4me3 have been identified as significant sites at which repressive and permissive modifications, respectively, occur in T cells [231,232,233]. As T cells differentiate into effector and memory T cells, the repressive H3K27me3 tends to be lost, while the permissive H3K4me3 modification is acquired [231,232].

### 7.3. Metabolic Intermediates Involved in Epigenetics

Emerging evidence suggests that intermediates from major metabolic pathways, including glycolysis, TCA cycle, fatty acid oxidation, and glutaminolysis, meet cell energy needs and provide intermediary metabolites that serve as substrate cofactors or inhibitors for epigenetic enzymes. These metabolites that drive epigenetic enzymes play crucial roles in orchestrating transcriptional and epigenetic programs and contribute to T cell fate and function. As mentioned above, acetyl-CoA serves as a critical source of the acetyl groups utilized by HATs [216]. At the same time, acetyl-CoA is primarily produced in the mitochondrial matrix in the context of multiple catabolic pathways, including oxidative metabolism of pyruvate (from glycolysis), FFAs, branched-chain amino acids, and ketone bodies in the TCA cycle [216]. To a lesser extent, acetyl-CoA is also produced in the cytosol, where it can play an essential role in multiple anabolic processes [216]. Thus, acetyl-CoA plays a crucial role in linking the metabolic status of the cell to the epigenetic modifications that dictate gene expression and the function of the cell. Additionally, within the TME, it has been demonstrated that upregulation of HIF-1a increases the production of the S enantiomer of 2-hydroxyglutarate (S-2HG), which is involved in driving epigenetic remodeling to enhance IL-2 production in antitumor T cells [129]. Recent studies suggest that culturing antitumor T cells with S-2HG ex vivo increases the central memory CD8^+^ population and enhances their antitumor efficacy [129,130].

Similarly, SIRTs require NAD^+^ to perform their deacetylase function, which plays a vital role in many cellular processes, including glycolysis, TCA cycle, and fatty acid oxidation [126]. Importantly, enhanced glycolysis has been linked to the decreased expression of SIRT1, which leads to the metabolic reprogramming of T cells to produce a highly cytotoxic subpopulation of CD8^+^ effector T cells [127]. Additionally, several recent investigations have also established the link between the availability of NAD^+^ and the dysfunctionality of T cells in the context of antitumor immunity [234]. A recent study showed that TILs possess two discrete chromatin states, one of which is a dysfunctional plastic state from which T cells can be rescued. The other is a fixed dysfunctional state that renders T cells resistant to reprogramming [235]. These discrete chromatin states correlate with specific surface protein expression profiles, defined as high CD38, CD5, and CD103 expression on the non-reprogrammable PD-1^hi^ dysfunctional T cells [235]. This is in line with our lab’s findings, which show that CD38^lo^ T cells, which are high in NAD^+^, control tumors despite PD-1 expression [93]. Another study showed that T cells that are unresponsive to B16-F10 melanoma exhibit low levels of Nampt, an enzyme involved in the de novo synthesis of NAD^+^ [236]. Using lung cancer and melanoma models, tumors treated with PD-1/PD-L1 blocking antibodies developed resistance through the upregulation of CD38 [237]. More recently, PD-1^+^CD38^+^ T cells were implicated in resistance to anti-PD-1 antibody treatment [238]. It is likely that the chromatin state of the T cells is regulated by CD38 expression, which is inversely correlated to NAD^+^ levels, and thus may be responsible for resistance to anti-PD-1 therapy [128]. Therefore, targeting CD38 expression on T cells may hold immense significance for the future of immunotherapy. Strategies that can selectively limit CD38 expression on T cells could reduce T cell dysfunctionality and could improve T cell-mediated tumor control.

## 8. Clinical Trials Targeting Immunometabolism

Despite the demonstrated importance of T cell metabolism in antitumor immune response in preclinical models, relatively few clinical trials have been conducted to investigate metabolic interventions in improving cancer immunotherapies aside from targeting IDO metabolism. As mentioned above, one of the most extensively studied interventions in clinical trials to date has been IDO inhibitors, which primarily work by blocking the conversion of tryptophan to kynurenine. IDO inhibitor trials have been initiated on a wide range of cancer types and stages and have met the intermediate successes described in Table 4. Other therapies targeting immunosuppressive metabolites, such as A2a receptor antagonists, glutaminase inhibitors, and therapies targeting CD73, CD39, and CD38, have also been investigated in Phase I/II clinical trials. Due to the relatively recent development of these targets, most clinical trials targeting them are still in the early stages, with unpublished or inconclusive results. Still, the results that have been reported so far demonstrate a favorable safety profile and early efficacy in small cohorts. Study status and/or outcomes are tabulated in Table 4. The results of these studies will be followed closely with great anticipation by the field. In addition to these strategies targeting immunosuppressive metabolites, several clinical trials are being conducted to target IL-7, IL-12, IL-15, and IL-21 cytokine signaling, as highlighted above in Table 3. To date, therapies targeting IL-7 and IL-15 have shown the most promise in improving the antitumor immune response with overall tolerable safety profiles. Conducting future clinical trials to optimize and/or combine these therapies will be necessary.

## 9. Conclusions and Future Directions

Cellular metabolism is considered a common thread that links gene transcription, signaling, function, and cell longevity. Strategies and efforts are being directed to understand how manipulating metabolic pathways and metabolite availability can alter cellular processes, particularly immune cells, to augment the antitumor immune response. While many of the studies mentioned above have shown promise, efforts to use these approaches for improving tumor control in human patients are currently limited in scope, as highlighted in the clinical trials presented in Table 3 and Table 4. Immune cell metabolism is highly dynamic and depends on various factors, including metabolite availability, cytokine signaling, and epigenetic modifications. This is especially true in antitumor immunity, in which T cells are exposed to unique metabolic conditions within the TME. Each of these previous efforts noted in this review has helped to improve our understanding of the dynamic changes in cellular metabolism that occur in T cell differentiation and function.

Future studies are needed to establish how immunometabolic approaches interact with established immunotherapies, including immune checkpoint blockade, and how these strategies can be combined. For example, our lab has previously demonstrated how decreasing the levels of S1P by inhibiting sphingosine kinase-1 can be combined with anti-PD-1 therapy to produce an enhanced anti-tumor response in preclinical models [34]. Other groups have demonstrated enhanced efficacy when combining therapies targeting metabolites [193] or cytokine signaling [183] with immune checkpoint blockade therapy. These preclinical studies have paved the way for several clinical trials (described above) utilizing combination strategies that have shown promising results so far.

In addition to combining immunometabolic approaches with existing immunotherapies, it may also be essential to identify common metabolic targets that can boost not only anti-tumor T cells but that also keep suppressive Tregs, myeloid-derived suppressor cells, and tumor-associated macrophages in check. Similarly, it will be essential to investigate the engagement of other immune cells, such as natural killer cells, gamma delta T cells, and innate lymphoid cells, by modulating their metabolism to provide an additional layer of targets that could be metabolically modulated for boosting tumor control. Such strategies hold significant promise and could provide robust tumor control in actual clinical settings in the future.

Despite the promise of these approaches, challenges still exist in translating immunometabolic strategies to the clinic. As is the case with immunotherapies in general, one of the key challenges is selectively enhancing the antitumor immune response without producing the systemic toxicities that are associated with a heightened immune response, such as autoimmunity and cytokine release syndrome. One potential strategy to address this challenge is the selective targeting pharmacological agents to the TME. For example, several groups have investigated the use of next-generation pro-cytokines that are activated specifically within the TME, as recently demonstrated by Guo et al. using a tumor-conditional IL-15 pro-cytokine to enhance memory development and antitumor efficacy while minimizing systemic toxicity [164]. Alternatively, the use of oncolytic viruses engineered to enforce expression of metabolic reprogramming agents has shown promise, as demonstrated Rivadeneira et al. with the use of a leptin-expressing oncolytic virus to selectively express leptin within the TME to metabolically reprogram TILs and to enhance tumor control [81]. Additional strategies to specifically enhance the immunometabolism of antitumor immune cells without promoting immunosuppressive immune cells or enhancing tumor metabolism will lead to improved outcomes for patients.

## Figures and Tables

**Table 1:** Novel metabolite targets in antitumor immunometabolism.

Metabolite	Role in Antitumor Immune Response	Potential Therapeutic Strategies	Ref.
Fatty acids	Fatty acid oxidation promotes and sustains memory T cell development while also supporting the immunosuppressive function of Tregs	Upregulate or overexpress fatty acid transporters and enzymes involved in FAO in antitumor T cells while inhibiting fatty acid uptake in Tregs	[119]
S1P	S1P promotes Treg development via PPARγ and contributes to limited antitumor immune response	Inhibit sphingosine kinase-1 activity in antitumor T cells and Tregs	[34]
L-arginine	Increased L-arginine in activated T cells shifts metabolism from glycolysis to OXPHOS, increasing central memory T cells and promoting antitumor activity	Increase extracellular L-arginine levels and increase L-arginine cellular transporters	[36]
Serine	Extracellular serine is necessary to support optimal T cell activation and proliferation	Increase extracellular serine levels and increase L-arginine cellular transporters	[37]
Lactate	Lactate impairs T cell activation and function, especially blunting activation of NFAT and production of IFNγ	Reduce lactate production by targeting lactate dehydrogenase A (LDHA)	[49,51]
Kynurenine	Kynurenine is produced by tumors cells, TAMs, and MDSCs via the enzyme IDO, inhibiting proliferation and effector function of effector T cells and inducing the expansion of Tregs	Block the production of kynurenine by inhibiting IDO activity	[61,63,64,65,68]
Tryptophan	Tryptophan is a key nutrient supporting antitumor T cell expansion and effector function. Depletion of tryptophan by IDO deprives T cells of this crucial nutrient and induces a decrease in global protein synthesis, and ultimately leads to T cell anergy	Block the consumption of tryptophan by tumor cells, TAMs, and MDSCs by inhibiting IDO activity	[67]
Adenosine	Adenosine interacts with the A2a receptor on effector T cells, inhibiting TCR signaling and expression of the IL-2 receptor while also upregulating the expression of immune checkpoint molecules	Decrease adenosine signaling or overall adenosine levels in the TME by targeting the A2a receptor or inhibiting CD39/CD73, the enzymes responsible for converting ATP to adenosine	[70,71]
Glutamine	Glutamine supports cancer cell growth while restricting glucose utilization and glycolysis in antitumor T cells, leading to metabolic dysfunction	Restrict glutamine uptake in the TME using inhibitors of glutamine transport	[74,116]
Acetate	Acetate promotes histone acetylation and chromatin accessibility in TILs and enhances IFNγ gene transcription and cytokine production in an acetyl-CoA synthetase (ACSS)-dependent manner	Supplement TILs with acetate and/or overexpress acetyl-CoA synthetase (ACSS)	[82]
NAD^+^	NAD+ serves as a key substrate for the histone deacetylase Sirt1 and contributes to post-translational modifications and epigenetic stability that lead to fit antitumor memory T cells metabolically	Increase intracellular levels of NAD^+^ in antitumor T cells, for example, by targeting CD38 expression or by programming T cells to have a hybrid Th1/17 phenotype	[93,126,127,128]
Acetyl-CoA	Acetyl-CoA is transferred by HATs for histone acetylation, epigenetically programming both antitumor T cells and Tregs	Determine how acetyl-CoA-producing metabolic processes can be altered to optimize epigenetic programming of antitumor T cells	[119]
S-2HG	S-2-hydroxyglutarate drives epigenetic remodeling to enhance IL-2 production in antitumor T cells	Culture antitumor T cells with S-2HG ex vivo to increase the central memory CD8^+^ population and enhance their antitumor efficacy	[129,130]

**Table 2:** Pre-clinical in vivo cancer models targeting metabolites and cytokines immunometabolism.

Target	Model	Strategy	Outcome	Ref.
Glycolysis	Murine melanoma	Activate adoptively transferred T cells in the presence of 2-deoxyglucose (glycolysis inhibitor)	Inhibition of glycolysis enhanced generation of memory and antitumor efficacy	[22]
Glycolysis/ FAO	Murine viral infection	Systemic administration of rapamycin to inhibit glycolysis and promote FAO	Systemic treatment with rapamycin enhanced memory T cell development and antitumor efficacy	[32]
Lipids (AGK)	Murine melanoma and colon cancer	Increase AGK activity in antitumor T cells	AKG-triggered PTEN inactivation promote glycolytic fitness and antitumor efficacy in CD8+ T cells	[186]
Lipids (S1P)	Murine melanoma	Pharmacological inhibition of sphingosine kinase 1 to decrease levels of S1P	Inhibition of sphingosine kinase 1 improved metabolic fitness and efficacy of antitumor T cells	[34]
L-arginine	Murine melanoma	Supplement antitumor T cells with L-arginine prior to ACT	L-arginine promoted a shift from glycolysis to OXPHOS and enhanced memory formation and antitumor efficacy	[36]
Mitochondrial metabolism	Murine melanoma	Selective transfer antitumor T cells with low mitochondrial membrane potential for ACT	ACT using T cells with low mitochondrial membrane potential resulted in superior antitumor efficacy	[38]
Mitochondrial metabolism	Murine melanoma	Enforce expression of PGC1a in T cells prior to ACT to enhance mitochondrial fitness	Enforced expression of PGC1a enhanced the ability of T cells to control tumor control	[80]
Thiols	Murine melanoma	Selective transfer antitumor T cells with high expression of surface thiols or treatment of T cells with thiol donors for ACT	Antitumor T cells expressing high levels of surface thiols demonstrated superior persistence and tumor control	[39]
Thioredoxin	Murine melanoma	Treatment of antitumor T cells with recombinant thioredoxin for ACT	Pre-treatment of T cells with thioredoxin resulted in enhanced persistence and tumor control upon ACT	[41]
Lactate	Murine melanoma	Reduce the activity of LDHA in tumor cells to decrease intratumoral levels of lactate	Reducing tumor cell production of lactate resulted in enhanced T cell infiltration and tumor control	[49]
Kynurenine	Mouse and canine melanoma, sarcoma, and breast cancer	Inhibit IDO enzyme activity in tumors to decrease levels of kynurenine and increase levels of tryptophan	Inhibition of IDO resulted in enhanced antitumor activity of CD8+ T cells and reduced frequencies of Tregs in the TME	[187]
Adenosine	Murine melanoma, colon, sarcoma, and breast cancer	Reduce intratumoral levels of adenosine by pharmacological inhibition of CD39/CD73 activity	Inhibition of CD39/CD73 ectonucleotidase activity decreased intratumoral levels of adenosine and enhanced the antitumor T cells response across multiple tumor models	[188,189,190,191,192]
Adenosine	Murine melanoma and head and neck cancer	Antagonize the A2AR receptor to enhance antitumor efficacy	Blockade of the A2AR resulted in enhanced tumor control alone and in combination with anti-PD-1 blockade	[193,194,195]
Leptin	Murine melanoma	Enhance the metabolic fitness of TILs using leptin-expressing oncolytic virus	Leptin expression in the TME reprogrammed TIL metabolism to promote tumor control	[81]
Acetate	Murine melanoma and lymphoma	Treat exhausted TILs with acetate to rescue effector function	Treatment of TILs ex vivo with acetate rescued their effector function and improved tumor control upon ACT	[82]
NAD+	Murine melanoma	Increased intracellular NAD+ levels in antitumor T cells by blocking CD38	Combining anti-CD38 antibody therapy with ACT resulted in enhanced tumor control and prolonged T cell survival	[93]
S-2HG	Murine lymphoma	Culture T cells with S-2HG to enhance IL-2 production and antitumor efficacy	Treatment of CAR-T ex vivo in the presence of S-2HG enhanced memory formation and antitumor efficacy	[130]
IL-15	Murine melanoma and lymphoma	Selectively increase intratumoral levels of IL-15 using tumor-conditional pro-IL-15	Use of tumor-conditional pro-IL-15 resulted in enhanced memory development and antitumor efficacy while minimizing systemic toxicity	[164]
IL-7	Murine neuroblastoma and glioblastoma	Engineer CAR-T cells to express a constitutively active IL-7 receptor	Expression of constitutively active IL-7 receptor enhanced the persistence and antitumor efficacy of CAR-T cells	[174]
IL-12	Murine melanoma	Condition antitumor T cells ex vivo with IL-12 prior to ACT	Ex vivo IL-12 conditioning enhanced in vivo expansion, proliferation, and antitumor efficacy of CD8^+^ T cells	[176]
IL-21	Murine melanoma	Condition antitumor T cells ex vivo with IL-21 prior to ACT	Ex vivo IL-21 conditioning enhanced the capacity of T cells to mediate tumor regression upon ACT	[181]
IL-10	Murine melanoma and colon cancer	Peritumoral administration of half-life-extended interleukin-10–Fc fusion protein, alone and in combination with anti-PD-1 therapy	IL-10–Fc fusion protein induced expansion of terminally exhausted TILs, upregulating OXPHOS and enhancing antitumor capacity of TILs	[185]

**Table 3:** Clinical trials targeting IL-7, IL-12, IL-15, and IL-21 cytokine signaling in immunotherapy.

Clinical Trial	Phase	Intervention	Cancer Type	Outcomes	Status
NCT05103631	I	IL-15 + GPC3-CAR-T cells	Hepatocellular carcinoma	N/A	Not yet recruiting (2021)
NCT04715191	I	IL-15 + IL-21 + GPC3-CAR-T cells	Hepatocellular carcinoma	N/A	Not yet recruiting (2021)
NCT04628780	I	anti-PD-1 targeting IL-15 fusion protein (PF07209960)	Advanced solid tumors	N/A	Recruiting (2021)
NCT04377932	I	IL-15 + GPC3-CAR-T cells	Hepatocellular carcinoma	N/A	Recruiting (2021)
NCT04294576	I	IL-15 fusion protein (BJ-001) subcutaneous injection + anti-PD-1 or anti-PD-L1 antibodies	Advanced solid tumors	BJ-001 is well tolerated up to 6 µg/kg [196].	Recruiting (2020)
NCT04261439	I	IL-15 (NIZ985) subcutaneous injection + anti-PD-1 antibody (spartalizumab)	Melanoma, advanced solid tumors	N/A	Recruiting (2021)
NCT03815682	I	Autologous multi-clonal T cell product loaded with IL15-Fc nanogel (RPTR-147) +/− pembrolizumab	Solid tumors, lymphoma	In a cohort of 17 patients, no dose-limiting toxicities were observed. A dose-dependent increase in inflammatory cytokines and CD8^+^ TILs was observed [197].	Recruiting (2021)
NCT03721068	I	Autologous iC9.GD2.CAR.IL-15 T-cells + chemotherapy	Neuroblastoma, osteosarcoma	N/A	Recruiting (2021)
NCT03388632	I	Recombinant IL-15 subcutaneous injection + nivolumab (anti-PD-1) or ipilimumab (anti-CTLA-4)	Metastatic solid tumors	N/A	Recruiting (2021)
NCT03127098	I/II	IL-15 (ALT-803) + ETBX-011 vaccine	Advanced CEA-expressing cancers	N/A	Completed (2019)
NCT02652910	I/II	IL-7/IL-15-programmed anti-CD19 CAR-T cells	Lymphoma	In a cohort of 18 patients, IL-7/IL-15-programmed anti-CD19 CAR-T cells offered substantial clinical benefit for NHL patients with manageable toxicities. ORR was 72.2% with complete remission of 36.7%. One case of CRS over grade 3 was observed [198].	Unknown (2019)
NCT02559674	I	IL-15 (ALT-803) + chemotherapy	Pancreatic cancer	N/A	Completed (2020)
NCT02452268	I	IL-15 (NIZ985) subcutaneous injection + anti-PD-1 antibody (PDR001)	Metastatic and advanced solid tumors	Combination therapy was well tolerated in pts with advanced solid tumors. No DLTs were observed [199].	Active, not recruiting (2021)
NCT02138734	I/II	Intravesical IL-15 (ALT-803) + BCG cancer vaccine	Non-muscle invasive bladder cancer	Combination therapy was well-tolerated. All patients were disease-free 24 months following combination therapy [200].	Recruiting (2021)
NCT01946789	I	IL-15 (ALT-803)	Advanced solid tumors	N/A	Completed (2019)
NCT01727076	I	Recombinant IL-15 subcutaneous injection	Advanced melanoma, kidney cancer, NSCLC, squamous cell head and neck cancer	Treatment was well tolerated. Substantial increases in circulating NK and CD8+ T cells was observed. A total of 2 SAEs were observed out of 19 [201].	Completed (2017)
NCT01572493	I	Recombinant IL-15 subcutaneous injection	Advanced cancers	8 SAEs observed out of 27 patients. Significant expansion of circulating CD8+ T cells and NK cells was observed [202].	Completed (2021)
NCT01369888	I/II	IV IL-15 + TIL infusion	Metastatic melanoma	Study was terminated due to autoimmune toxicity.	Terminated (2015)
NCT01189383	I/II	Autologous dendritic cells manufactured with GM-CSF and IL15 and loaded with melanoma/HIV peptides and KLH.	Metastatic melanoma	N/A	Completed (2016)
NCT01021059	I	Recombinant IL-15 IV injection	Melanoma, renal cell carcinoma	Treatment could be safely administered (0.3 μg/kg). Dose limiting toxicities were observed in patients receiving higher doses (3.0 and 1.0 μg/kg). Clearance of lung lesions was observed in two patients. Significant expansion of NK and memory CD8^+^ T cells was observed [203]	Completed (2019)
NCT01265368	I/II	Allogenic tumor cell vaccine expressing IL-7, GM-CSF, CD80, and CD154	Renal cell cancer	MGN1601 treatment was safe and feasible and resulted in improved cellular immune function with preliminary clinical efficacy [204]	Completed (2018)
NCT03198546	I	IL-7- and CCL19-secreting GPC3-specific CAR-T cells	GPC3+ HCC	IL-7 and CCL19-secreting CAR-T cells significantly enhanced antitumor activity, and the therapy was well-tolerated in a cohort of six patients [205]	Recruiting (2020)
NCT04099797	I	GD2-specific CAR with constitutively active IL-7 receptors ((C7R)-GD2.CART)	High grade glioma, diffuse intrinsic pontine glioma	N/A	Recruiting (2021)
NCT03635632	I	GD2-specific CAR with constitutively active IL-7 receptors ((C7R)-GD2.CART)	Refractory neuroblastoma and other GD2+ cancers	N/A	Recruiting (2021)
NCT02960594	I	IL-12 DNA plasmid (INO-9012) + hTERT vaccine (INO-1400 or INO-1401)	Advanced solid tumors	N/A	Completed (2018)
NCT04911166	I	Adenoviral-mediated interleukin-12 gene therapy +/− atezolizumab	NSCLC	N/A	Recruiting (2021)
NCT04756505	I	NHS-IL-12 + bispecific anti-PD-1/TGFβ antibody (bintrafusp alfa)	Hormone receptor positive HER2 negative breast cancer	N/A	Recruiting (2021)
NCT01236573	I/II	IL-12 gen-transduced TILs + chemotherapy	Metastatic melanoma	Terminated due to unexpected toxicities, likely due to TIL product and the low percentage of durable responses.	Terminated (2019)
NCT02062827	I	Genetically engineered IL-12-expressing oncolytic herpes simplex virus (M032)	Glioblastoma multiforme	N/A	Recruiting (2021)
NCT00347971	I	Recombinant IL-21 + rituximab	Non-Hodgkin lymphoma	Clinical responses were seen in 8 of 19 evaluable patients. Durable complete remission was observed in a small subset of patients. Therapy was well-tolerated [206]	Completed (2008)
NCT00389285	I/II	Recombinant IL-21 +/− sorafenib	Renal cell carcinoma	ORR was 21% and disease control rate was 82% with combination therapy. In phase II, the therapy was well-tolerated with toxicities being mostly graded 1 or 2 [207]	Completed (2009)

**Table 4:** Clinical trials targeting immunosuppressive metabolites in immunotherapy.

Clinical Trial	Phase	Intervention	Cancer Type	Outcomes	Status
NCT01219348	I	IDO peptide vaccination + imiquimod	NSCLC	Long-lasting disease stabilization without toxicity [239]	Completed 2015
NCT03047928	I/II	IDO peptide vaccination + anti-PD-1 antibody	Metastatic melanoma	Systemic toxicity profile was comparable to nivolumab monotherapy; objective response rate of 80% was reached.	Recruiting (2020)
NCT01685255	II	IDO inhibitor, (epacadostat, INCB024360)	Ovarian cancer	Study terminated due to slow accrual and lack of evidence of superiority [240]	Terminated
NCT01792050	II	IDO inhibitor (indoximod, NLG-8186) + Taxane chemotherapy	Metastatic breast cancer	Addition of indoximod to a taxane did not improve PFS compared with a taxane alone [241]	Completed (2020)
NCT03343613	I	IDO-1 inhibitor (LY3381916) + anti-PD-L1 antibody	NSCLC, renal cell carcinoma, breast cancer	LY3381916 is safely administered as monotherapy and in combination with anti-PD-L1 therapy [242]	Terminated (2020)
NCT04106414	II	IDO inhibitor (BMS-986205) + anti-PD-1 antibody	Endometrial Adenocarcinoma	N/A	Active, not recruiting (2021)
NCT03915405	I	IDO inhibitor (KHK2455) + anti-PD-L1 antibody	Urothelial carcinoma	N/A	Recruiting (2021)
NCT02052648	I/II	IDO inhibitor (indoximod, NLG-8186) + chemotherapy or Bevacizumab	Recurrent glioma	N/A	Completed (2020)
NCT03164603	I	IDO inhibitor (NLG802)	Solid tumors	N/A	Completed (2020)
NCT02073123	I/II	IDO inhibitor (indoximod, NLG-8186) + immune checkpoint inhibition	Metastatic Melanoma	Combination of indoximod and pembrolizumab demonstrated an ORR of 55.7%, CR 18.6%, which compares favorably with reported ORR for pembrolizumab alone [243]	Completed (2020)
NCT02048709	I	IDO1 inhibitor (Navoximod, GDC-0919)	Solid tumors	Navoximod was well-tolerated and decreased plasma kynurenine levels. Stable disease responses were observed [244]	Completed (2017)
NCT02077881	I/II	IDO inhibitor (indoximod, NLG-8186) + chemotherapy	Metastatic pancreatic cancer	Generally well-tolerated. OS of 10.9 months and ORR of 46.2%. Increased intra-tumoral CD8 density was observed [245]	Completed (2020)
NCT02502708	I	IDO inhibitor (indoximod, NLG-8186) + chemotherapy	Pediatric brain tumors	Combining indoximod with chemotherapy was well tolerated with improved outcomes [246]	Completed (2020)
NCT02460367	I	IDO inhibitor (indoximod, NLG-8186) + Tergenpumatucel-L	NSCLC	N/A	Active, not recruiting (2020)
NCT03414229	II	IDO inhibitor (epacadostat, INCB024360) + anti-PD-1 antibody	Sarcoma	Epacadostat + pembrolizumab was well tolerated but had limited anti-tumor activity [247]	Active, not recruiting (2021)
NCT02166905	I/II	IDO inhibitor (epacadostat, INCB024360) + DEC-205/NY-ESO-1 fusion protein CDX-1401 + poly ICLC	Fallopian tube carcinoma, ovarian carcinoma, peritoneal carcinoma	N/A	Completed (2021)
NCT03896113	II	Celecoxib	Endometrium cancer	N/A	Recruiting (2020)
NCT01961115	II	IDO inhibitor (epacadostat, INCB024360)	Melanoma	Epacadostat therapy was considered safe with transient DLTs in only two patients. Pacadostat normalized serum Kyn/Trp ratios in 91% of patients. Clinical activity was observed. Enhanced CD8 T cell infiltration was observed.	Completed (2018)
NCT03491631	I	IDO inhibitor (SHR9146) + apatinib	Solid tumors	SHR9146 plus apatinib demonstrated promising anti-tumor activity with acceptable safety profile [248]	Completed (2018)
NCT02118285	I	IDO inhibitor (epacadostat, INCB024360) + IP NK cells + IL-2	Fallopian tube carcinoma, ovarian carcinoma, peritoneal carcinoma	N/A	Completed (2017)
NCT03459222	I/II	IDO inhibitor (BMS-986205) + Relatlimab + Nivolumab	Advanced solid cancers	N/A	Recruiting (2021)
NCT04047706	I	IDO inhibitor (BMS-986205) + radiation + nivolumab +/− temozolomide	Glioblastoma	N/A	Recruiting (2020)
NCT03361865	III	IDO inhibitor (epacadostat, INCB024360) + pembrolizumab	Urothelial cancer	ORR of 31.8% in treatment group compared to 24.5% in placebo group. Serious adverse events detected in 30.23% of treatment group compared to 26.53% of placebo group.	Completed (2020)
NCT03358472	III	IDO inhibitor (epacadostat, INCB024360) + pembrolizumab or EXTREME regimen	Head and neck cancer	ORR of 31.4% in combination group compared to 21.1% in pembrolizumab and 34.3% in EXTREME regimen. Similar rates of serious adverse events in all groups.	Active, not recruiting (2021)
NCT03322540	II	IDO inhibitor (epacadostat, INCB024360) + pembrolizumab	Lung cancer	ORR of 32.5% in combination group compared to 39.0% with pembrolizumab alone. Similar rates of serious adverse events in all groups.	Completed (2021)
NCT03260894	III	IDO inhibitor (epacadostat, INCB024360) + pembrolizumab	Renal cell carcinoma	N/A	Active, not recruiting (2020)
NCT03322566	II	IDO inhibitor (epacadostat, INCB024360) + pembrolizumab + chemotherapy	NSCLC	ORR of 26.4% in combination group compared to 44.8% in group receiving pembrolizumab + chemotherapy + placebo.	Completed (2021)
NCT03006302	II	IDO inhibitor (epacadostat) + pembrolizumab + cyclophosphamide +/− GVAX pancreas vaccine	Metastatic pancreatic adenocarcinoma	N/A	Recruiting (2021)
NCT05106296	I	IDO inhibitor (indoximod, NLG-8186) + chemotherapy + ibrutinib	Ependymoma, medulloblastoma, glioblastoma, primitive neuroectodermal tumor (PNET)	N/A	Not yet recruiting (2021)
NCT04049669	II	IDO inhibitor (indoximod, NLG-8186) + chemotherapy + radiation	Ependymoma, medulloblastoma, glioblastoma, diffuse intrinsic pontine glioma	N/A	Recruiting (2021)
NCT03347123	I/II	IDO inhibitor (epacadostat) + nivolumab + ipilimumab or lirilumab	Solid tumors	N/A	Completed (2021)
NCT03085914	I/II	IDO inhibitor (epacadostat) + pubmrolizumab + chemotherapy	Solid tumors	N/A	Completed (2021)
NCT03207867	II	A2a receptor antagonist (NIR178) + anti-PD-1 antibody	Solid tumors, diffuse large B-cell lymphoma	N/A	Recruiting (2021)
NCT03381274	I/II	A2a receptor antagonist (AZD4635) + anti-CD73 antibody (MEDI9447) or Osimertinib	NSCLC	N/A	Active, not recruiting (2021)
NCT02403193	I/II	A2a receptor antagonist (PBF-509) +/− anti-PD-1 antibody	NSCLC	NIR178 was well tolerated. Clinical benefit was observed in patients irrespective of PD-L1 status [249]	Active, not recruiting (2020)
NCT04089553	II	A2a receptor antagonist (AZD4635) + anti-CD73 antibody (oleclumab) or anti-PD-L1 antibody (Durvalumab)	Prostate cancer	N/A	Active, not recruiting (2021)
NCT02740985	I	A2a receptor antagonist (AZD4635) +/− anti-CD73 antibody (oleclumab), anti-PD-L1 antibody (durvalumab), or chemotherapy	NSCLC, prostate cancer, colorectal carcinoma	AZD4635 monotherapy or in combination with durvalumab displayed a tolerable safety profile and was associated with clinical benefit [250]	Active, not recruiting (2021)
NCT03267589	II	anti-CD73 antibody (MEDI9447) +/− anti-PD-1 antibody or anti-CTLA4 antibody	Ovarian cancer	N/A	Recruiting (2021)
NCT02754141	I/II	anti-CD73 antibody (BMS-986179) +/− anti-PD-1 antibody (nivolumab) or rHuPH20	Advanced solid tumors	BMS-986179 + nivolumab combination therapy was well tolerated with similar safety profile compared to nivolumab alone. Combination therapy demonstrated preliminary antitumor efficacy [251]	Active, not recruiting (2021)
NCT03549000	I	anti-CD73 antibody (NZV930) +/− A2a receptor antagonist (NIR178) or anti-PD-1 antibody	Advanced cancers	N/A	Recruiting (2021)
NCT04969315	I/II	A2a receptor antagonist (TT-10)	Renal cell cancer, castrate-resistant prostate cancer, NSCLC	N/A	Not yet recruiting (2021)
NCT02655822	I	A2a receptor antagonist (ciforadenant) +/− anti-PD-L1 antibody (atezolizumab)	Renal cell cancer, Prostate cancer, NSCLC	Ciforadenant is well tolerated and showed anti-tumor activity as monotherapy and in combination atezolizumab for patients with renal cell cancer and NSCLC	Completed (2021)
NCT04797468	I	CD73 inhibitor (HLX23)	Advanced solid tumors	N/A	Not yet recruiting (2021)
NCT05143970	I	anti-CD73 antibody (IPH5301) +/− chemotherapy and trastuzumab	HER2+ cancers	N/A	Not yet recruiting (2021)
NCT04148937	I	CD73 inhibitor (LY3475070)	Advanced cancers	N/A	Active, not recruiting (2021)
NCT04572152	I	anti-CD73 antibody (AK119) + PD-1/CTLA-4 bispecific antibody	Advanced or metastatic solid tumors	N/A	Recruiting (2021)
NCT04672434	I	anti-CD73 antibody (Sym024) +/− anti-PD-1 antibody (Sym021)	Metastatic solid tumors	N/A	Recruiting (2021)
NCT05119998	I	anti-CD73 antibody (IBI325) +/− anti-PD-1 antibody (sintilima)	Solid tumors	N/A	Not yet recruiting (2021)
NCT04940286	II	anti-CD73 antibody (Oleclumab) + chemotherapy + anti-PD-L1 antibody (Durvalumab)	Pancreatic cancer	N/A	Recruiting (2021)
NCT03454451	I	anti-CD73 antibody (CPI-006) +/− A2a receptor antagonist (ciforadenant) or anti-PD-1 antibody (pembrolizumab)	Advanced cancers	Treatment is well-tolerated with early evidence of anti-tumor activity of CPI-006 monotherapy. Increased CD4+:CD8+ T cell ratio with CPI-006 therapy [252]	Recruiting (2021)
NCT02503774	I	anti-CD73 antibody (MEDI9447) +/− anti-PD-L1 antibody (durvalumab)	Solid tumors	Combination therapy demonstrated a tolerable safety profile with promising antitumor activity in EGFRm NSCLC [253]	Active, not recruiting (2021)
NCT05075564	I	anti-CD39 antibody (ES002023)	Advanced solid tumors	N/A	Not yet recruiting (2021)
NCT04306900	I	anti-CD39 antibody (TTX-030) +/− anti-PD-1 antibody and chemotherapy	Adult solid tumors	N/A	Recruiting (2021)
NCT04336098	I	anti-CD39 antibody (SRF617) +/− chemotherapy or anti-PD-1 antibody	Advanced solid tumors	N/A	Recruiting (2021)
NCT03884556	I	anti-CD39 antibody (TTX-030) +/− anti-PD-1 antibody or chemotherapy	Solid tumors, lymphoma	N/A	Recruiting (2021)
NCT03473730	I	anti-CD38 antibody (daratumumab)	Metastatic renal cell carcinoma, invasive bladder cancer	N/A	Active, not recruiting (2021)
NCT03177460	I	anti-CD38 antibody (daratumumab) +/− FMS Inhibitor	Prostate cancer	N/A	Active, not recruiting (2020)
NCT03637764	I/II	anti-CD38 antibody (isatuximab) +/− anti-PD-L1 (atezolizumab)	HCC, SCCHN, EOC	CD38 inhibition does not seem to influence response to anti-PD-L1 agents in these patients with HCC, SCCHN, or EOC [254]	Active, not recruiting (2021)
NCT03367819	I/II	anti-CD38 antibody (isatuximab) +/− anti-PD-1 (cemiplimab)	Prostate cancer, NSCLC	Combination therapy was associated with a tolerable safety profile, reduction of CD38+ T cells in the TME, and activation of peripheral T cells, but no significant antitumor activity was observed in these small cohorts [255]	Completed
NCT04265534	II	Glutaminase inhibitor (telaglenastat) + anti-PD-1 antibody (pembrolizumab) + chemotherapy	KEAP1/NRF2-mutated NSCLC	N/A	Active, not recruiting (2021)
